# Bacitracin resistance and enhanced virulence of *Streptococcus suis* via a novel efflux pump

**DOI:** 10.1186/s12917-019-2115-2

**Published:** 2019-10-28

**Authors:** Jiale Ma, Jin Liu, Yue Zhang, Dan Wang, Runxia Liu, Guangjin Liu, Huochun Yao, Zihao Pan

**Affiliations:** 1 College of Veterinary Medicine, Nanjing Agricultural University, Nanjing, 210095 China; 2 OIE Reference Laboratory for Swine Streptococcosis, Nanjing, 210095 China; 3 South Dakota State University, Brookings, SD 57007 USA; 4 MOE Joint International Research Laboratory of Animal Health and Food Safety, Nanjing, China

**Keywords:** Bacitracin, Virulence, Serotype Chz, *Streptococcus suis*, SstFEG, Efflux pump

## Abstract

**Background:**

*Streptococcus suis* is a prominent pathogen causing septicemia and meningitis in swine and humans. Bacitracin is used widely as a growth promoter in animal feed and to control the spread of necrotic enteritis in most developing countries. This study aimed to characterize a novel membrane transporter module Sst comprising SstE, SstF, and SstG for bacitracin resistance.

**Results:**

Comparative genomics and protein homology analysis found a potential efflux pump SstFEG encoded upstream of well-known bacitracin-resistance genes *bceAB* and *bceRS*. A four-fold decrease in bacitracin susceptibility was observed in *sstFEG* deletion mutant comparing with *S. suis* wildtype strain CZ130302. Further studies indicated that the bacitracin tolerance mediated by SstFEG is not only independent of the BceAB transporter, but also regulated by the two-component system BceSR. Given that SstFEG are harbored by almost all virulent strains, but not in the avirulent strains, we managed to explore its potential role in bacterial pathogencity. Indeed, our results showed that SstFEG is involved in *S. suis* colonization and virulence in animal infection model by its potential competitive survival advantage against host bactericidal effect.

**Conclusion:**

To our knowledge, this is the first study to functionally characterize the bacitracin efflux pump in *S. suis* to provide evidence regarding the important roles of the novel ABC transporter system SstFEG with respect to drug resistance and virulence.

## Background

*Streptococcus suis* is an emerging zoonotic pathogen imposing a serious burden on the porcine industry as well as a severe public health concern [1, 2]. It is found extensively in porcine breeding environments, which are considered as a potential natural reservoir of resistance genes among several bacteria [3–5]. Bacitracin produced by *Bacillus licheniformis* is a type of narrow-spectrum peptide acting on Gram-positive bacteria [6]. Bacitracin was previously used as a growth-promoting supplement in animal feed [7]. Furthermore, bacitracin can effectively control necrotic enteritis [8], which is used as a preparation in numerous countries. Prolonged use of bacitracin in animals increases resistance genes in microorganisms. Some molecular mechanisms underlying bacitracin resistance in bacteria have been reported [9–11].

In one such pathway, the Bce system comprising a two-component system (TCS) BceSR and the membrane transporter BceAB to export bacitracin [12]. Similar pathways have been identified in *Bacillus subtilis* [6]*, Streptococcus mutants* [13]*, Enterococcus faecalis* [9], and *Clostridium perfringens* [14]. Furthermore, *Escherichia coli* and *Staphylococcus aureus* highly express undecaprenol kinase to convert undecaprenol pyrophosphate to undecaprenol phosphate. Some organisms produce exopolysaccharides, such as *Streptococcus mutans*, or membrane-bound cell-surface phospholipids, such as *Xanthomonas campestris* [15], which could bind bacitracin to remain extracellular. Bce systems are widely distributed in bacteria as a bacitracin efflux pump. The Bce system functions as an importer, as revealed through structural studies. In Bce systems, the RS regulon functions as the key bacitracin-resistance locus; the other module AB pumps out bacitracin via a transmembrane channel. The present study aimed to characterize a novel membrane ABC transporter module Sst comprising three components SstF, SstE, and SstG, and their role in the regulation of bacitracin resistance. We generated a series of mutant strains to compare their differences in bacitracin resistance with the wild-type strain through a drug sensitivity test and qRT-PCR analysis for *bceRS*, *bceAB*, and *sstFEG* genes. Mice challenge experiments testing bacterial colonization and survival rate were further performed to assess the pathogenicity of the Sst FEG modules in strain CZ130302.

## Results

### A potential efflux pump for drug-resistance encoded by *sstFEG*

**Fig. 1:**
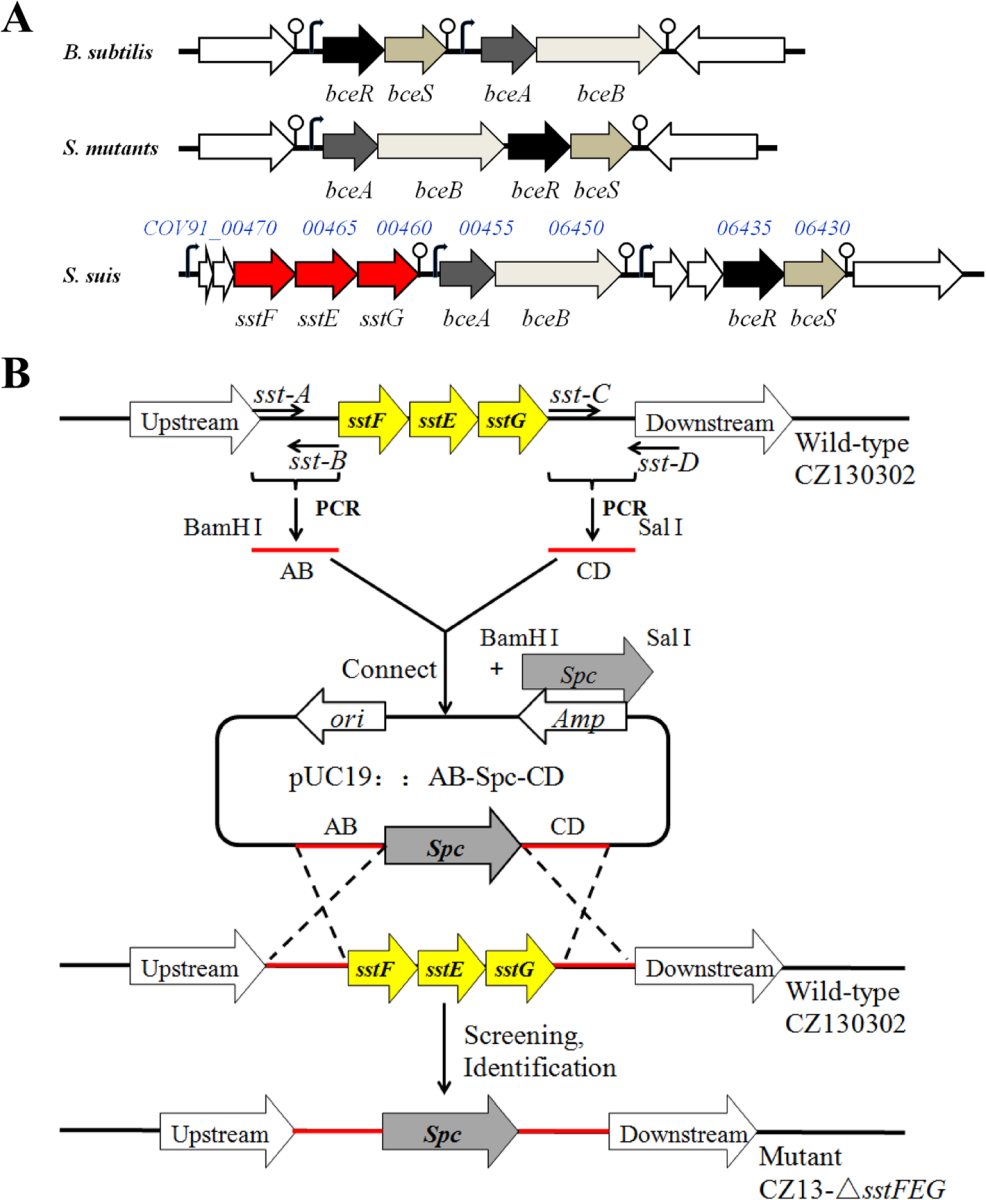
The genetic neighborhood analysis and the mutant construction strategy. **a** A schematic representation of the genetic locus containing seven genes (CVO91___06470 to CVO91___06430), along with the adjacent genes, comparing gene organization characteristics in *Bacillus subtilis*, *Streptococcus mutans*, and *S. suis*. The genes *bceABSR* are ubiquitous, while *sstFEG* are exclusively present in *S. suis*. Sequence analysis of this genetic locus indicates three potential transcriptional terminators in the region between *sstG* and *bceA* and downstream of *bceB* and *bceS.* **b** A schematic representation of mutant construction strategy. Three fragments AB, Spc, and CD were fused to form an intermediate vector with pUC19 plasmid. The mutant strains were obtained via natural genetic transformation, screening, and identification

Recently, we reported a highly virulent *S. suis* strain CZ130302 designated as the new serotype Chz causing acute meningitis in piglets. Unexpectedly, CZ130302 also displayed a more extensive drug resistance pattern than *S. suis* serotype 2 (SS2) reference strains P1/7 and HA9801 in the MIC test, including bacitracin (Table 1). Whole genome retrieval of CZ130302 (CP024974.1) identified a gene cluster encoding the well-known bacitracin transporter BceAB and the related TCS BceSR (Fig. 1a). Further genetic annotation and prediction revealed a novel ABC transporter located at the upstream of *bceAB*, which was predicted as a potential efflux pump for drug-resistance. Thus, three genes, CVO91_06470, CVO91_06465, and CVO91_06460, were designated as *sstFEG* for further studies.

**Table 1:** The MICs of CZ130302 to different kinds of antibiotics

Antibiotics	MIC(ng/μL)^a^	Antibiotics	MIC(ng/μL) ^a^
Penicillin G	16	Tetracycline	128
Ampicillin	64	Doxycycline	32
Streptomycin	256	Erythromycin	512
Gentamicin	256	Lincomycin	512
Kanamycin	> 512	Bacitracin	64
Spectinomycin	32	Vancomycin	0.25
Amikacin	128	Nisin	256
Neomycin	> 512	Ciprofloxacin	64
Chloramphenicol	4	Norfloxacin	128

^a^The *Streptococcus pneumoniae* ATCC 49619 was used as control strain here

### SstFEG mediates bacitracin tolerance in *S. suis*

**Fig. 2:**
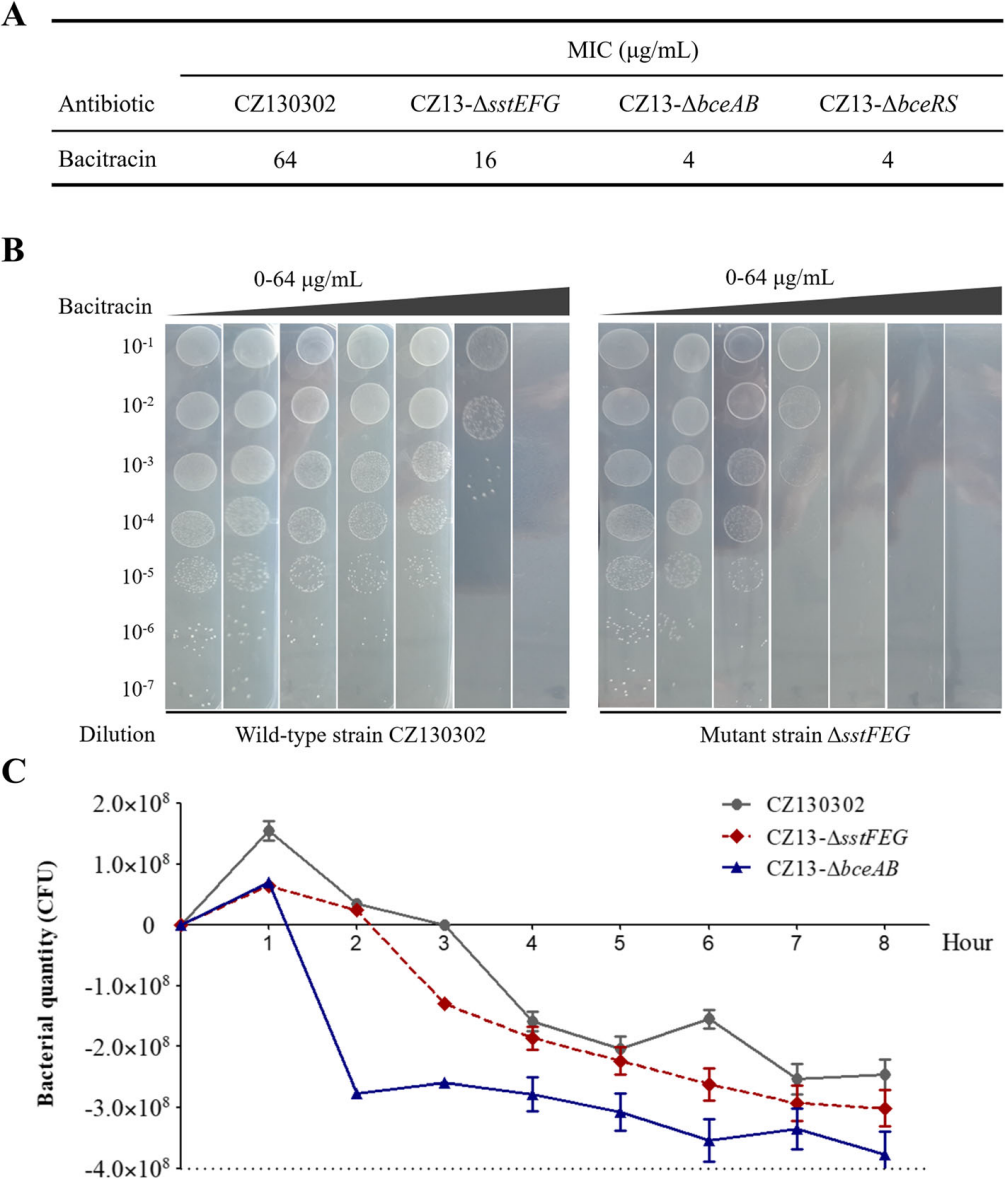
Bacitracin sensitivity of the mutant strains. **a** Comparison of MICs to bacitracin between wild-type and mutant strains. **b** The bacterial colony growth in THB plate with bacitracin resistance. Bacterial cells in the logarithmic phase were subjected to serial dilution and 10 μL of bacterial cultures were in THB plates supplemented with bacitracin at different concentrations (0, 2, 4, 8, 16, 32, and 64 μg/mL). **c** The bacterial survival of wild-type and mutant strains incubated with high bacitracin concentration. The bacterial cells were eliminated with 128 μg/mL bacitracin. We monitored and recorded the bacterial loads incubated after 8 h with 128 μg/mL bacitracin in the logarithmic phase. The CFU values of all mutants was reduced to a greater extent in comparison with the wild-type strain at each hour

Subsequently, a series of deletion mutants were constructed via homologous replacement with a spectinomycin (Spc) resistance expression cassette [16]. Construction of the mutant strains was confirmed via PCR (Additional file 1). The bacterial growth of CZ13-△*sstFEG* showed 1 h delay in log-phase compared with that of wild-type strain (Additional file 2), but no significant difference in the last stage. It should be noted that the bacitracin sensitivity of different mutant strains was increased at varying degrees in comparison with the wild-type strain (Fig. 2a). The mutant strain CZ13-△*sstFEG* (MIC, 16 μg/mL) was approximately 4-fold more sensitive to bacitracin than the wild-type CZ130302 strain was (MIC, 64 μg/mL), concurrent with the colony growth of CZ13-△*sstFEG* on the culture media supplemented with bacitracin at different concentrations (Fig. 2b). Otherwise, CZ13-△*bceAB* and CZ13-△*bceRS* (MIC, 4 μg/mL) were more sensitive to bacitracin than CZ13-△*sstFEG* was (MIC, 16 μg/mL). Based on the MIC, bacterial growth inhibition at 8 h was compared upon bacitracin supplementation at high concentrations in the log-phase. Obviously, the mutant strain CZ13-△*bceAB* displayed the greatest reduction in bacterial survival at each time point (Fig. 2c). In summary, both BceAB and SstFEG transporters co-regulate bacitracin resistance in strain CZ130302, but their functional correlation in this process is unclear.

### Bacitracin tolerance mediated by SstFEG is independent of the BceAB transporter

**Fig. 3:**
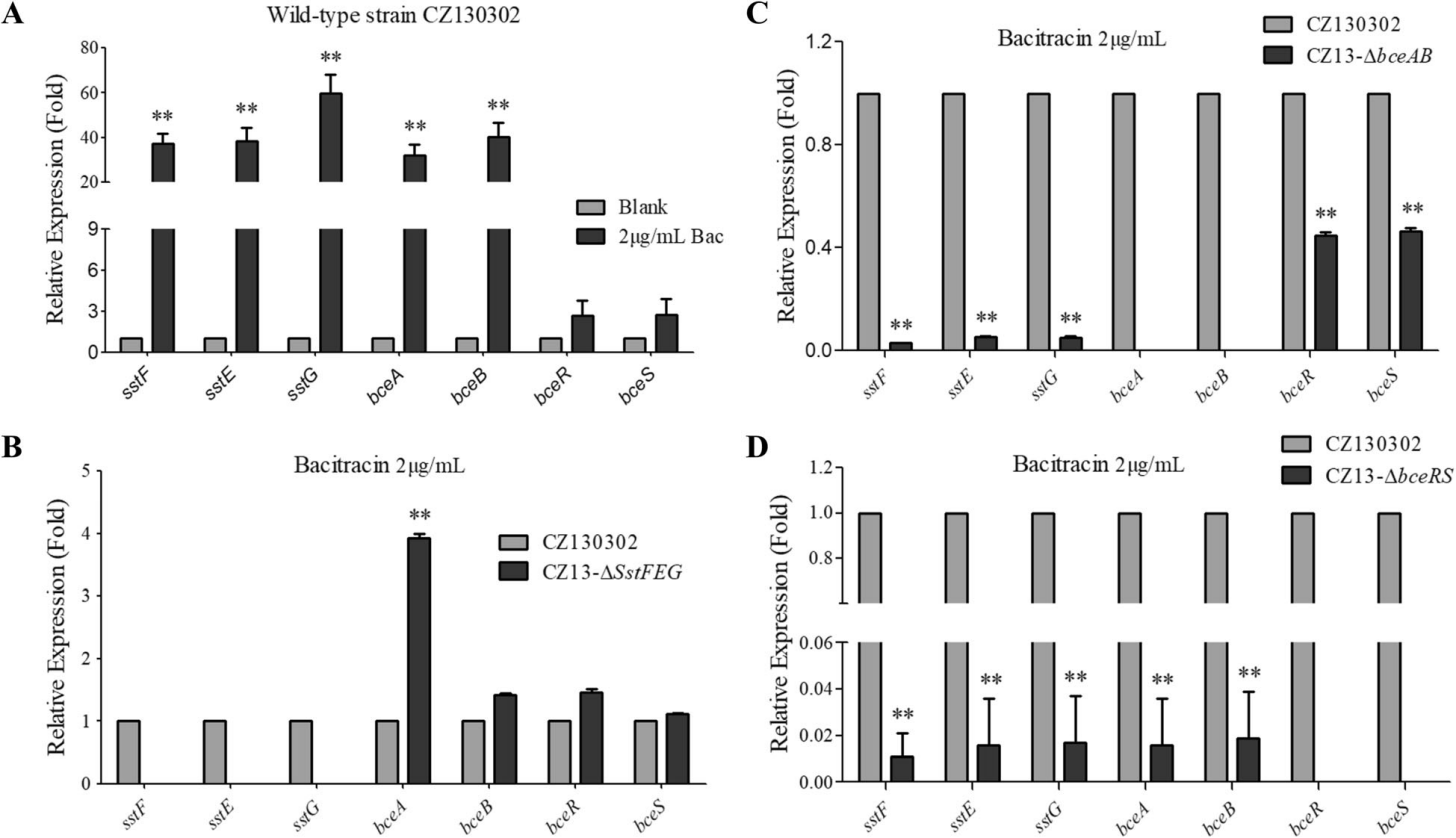
Real-time qRT-PCR analysis of the *sstFEG*, *bceAB*, and *bceRS*. The data were normalized to the housekeeping gene parC transcript [18]. The relative expression levels represent the mean ± SD for three independently isolated RNA samples. (**a**) The comparison of gene expression in the presence (Bac [2 μg/mL]) or absence of bacitracin. The concentration of bacitracin at 2 μg/mL was tested that could not kill S. suis cells rapidly. (**b**, **c**, **d**) The expression changes of *sstFEG*, *bceAB* and *bceRS* in different deletion mutant under the bacitracin stress. Several-fold changes represent the relative gene expression in the wild-type and mutant strains cultured in the presence (Bac [2 μg/mL]) of bacitracin

To further understand the functional characteristics of SstFEG, BceAB, and BceRS when bacterial cells responded to bacitracin stress, total RNAs of wild-type and mutant strains were extracted under the same culture conditions with or without bacitracin treatment, and the expression of relevant seven genes (*sstFEG*/*bceAB/bceRS*) were comparatively analyzed via qRT-PCR. As shown in Fig. 3a, the bacitracin supplementation in culture medium significantly activated the expression of *sstFEG* and *bceAB* at least 30-fold higher than that in the normal culture. However, the transcriptional level of *bceRS* was not significantly upregulated under this condition. These results indicated that the *bceRS* and *sstFEG/bceAB* were controlled by different operons, suggesting that the following study need to be performed under the bacitracin treatment to confirm the function of SstFEG in bacitracin resistance directly or indirectly.

Indeed, our previous genetic analysis showed that *sstFEG*, *bceAB*, and *bceRS* are controlled by different operons (Fig. 1a), respectively. Here, we need to confirm whether the deletion of *sstFEG* caused the expression change of *bceAB* and *bceRS,* which have been reported for bacitracin resistance in several bacterial species [12]. As shown in Fig. 3b, the deletion of *sstFEG* did not downregulate the expression of *bceABRS* and even caused the significant activation of *bceA* by more than 4-fold. These results suggested that the deficiency of bacitracin resistance caused by *sstFEG* deletion does not relate with well-known BceAB and BceSR systems. Unexpectedly, the deletion of *bceAB* significantly inhibited the expression of *sstFEG* (Fig. 3c), suggesting that the deficiency of bacitracin resistance caused by *bceAB* deletion may be related with BceAB inactivation and *sstFEG* downregulation together. These observations, together with the results shown in Fig. 2, further implied that SstFEG is an efflux pump for bacitracin and can transport bacitracin independently.

### BceSR regulates SstFEG in response to bacitracin resistance

Here, we explored why the deletion of *bceAB* inhibited the expression of *sstFEG* (Fig. 3c), and found that *bceRS* is downregulated by approximately 50% in the △*bceAB* mutant. As revealed from the KEGG Pathway Database (Additional file 3) and previous studies [11], BceAB transporter serves as an efflux pump along with the BceSR TCS to regulate bacitracin perception. Thus, it is reasonable to speculate that BceSR also is the important regulator of *sstFEG* operon. Indeed, the deletion of *bceRS* caused significant downregulation of *sstFEG* and *bceAB* more than 50-fold (Fig. 3d), which almost lost the transcriptional activation of these genes in response to bacitracin stress under the 2 ng/μL concentration, indicating that BceSR is the major regulator for bacitracin resistance in this case. These results suggested that the downregulation of *bceRS* caused by the deletion of *bceAB* may be the primary reason of *sstFEG* transcriptional inhibition, and *sstFEG* regulation by BceSR.

### *S. suis* colonization and virulence requires bacitracin tolerance

The prevalence of *sstFEG, bceAB*, and *bceSR* genes in 35 whole genomes of *S. suis* from NCBI database were investigated, which showed that all strains encode *bceAB* and *bceRS* genes, but only 19 strains (18 highly virulent or virulent strains except for T15) encode *sstFEG* genes, and almost all avirulent strains are *sstFEG* negative (Table 2). In particular, serotype 3 strain ST3, isolated from a pig with pneumonia in Hubei province in 2009 [43], only harbors *sstF*, but not *sstE* and *sstG*. These results suggested that SstFEG may be involved in the pathogenicity of *S. suis*.

**Table 2:** The distribution of seven bacitracin resistance genes in *Streptococcus suis*

Strains	Serotype^a^	Virulence	host	Symptoms	*BceB/A*	*BceS/R*	*sstFEG*	Reference
CZ130302	Chz	Highly virulent	piglet	Meningitis	+	+	+	[18]
BM407	2	Highly virulent	human	STSS	+	+	+	[19]
05ZYH33	2	Highly virulent	human	STSS	+	+	+	[20]
98HAH33	2	Highly virulent	human	STSS	+	+	+	[20]
GZ1	2	Highly virulent	human	STSS	+	+	+	[21]
SC84	2	Highly virulent	human	STSS	+	+	+	[19]
SC19	2	Highly virulent	piglet	Meningitis	+	+	+	[22]
05ZY719	2	Highly virulent	piglet	Septicemia	+	+	+	[23]
SC070731	2	Highly virulent	piglet	Meningitis	+	+	+	[24]
P1/7	2	Highly virulent	pig	Septicemia	+	+	+	[25]
S735	2	Highly virulent	pig	Septicemia	+	+	+	[26]
A7	2	Highly virulent	pig	Septicemia	+	+	+	[27]
CS100322	2	Virulent	pig	lung	+	+	+	[28]
SS2–1	2	Virulent	pig	diseased	+	+	+	[29]
T15	2	Avirulent	pig	Septicemia	+	+	+	[30]
SS12	1/2	Virulent	pig	lung	+	+	+	[27]
JS14	14	Highly virulent	pig	lung	+	+	+	[31]
GZ0565	9	Highly virulent	pig	Septicemia	+	+	+	[31]
LSM102	–	Highly virulent	pig	Septicemia	+	+	+	[32]
ST3	3	Virulent	pig	Septicemia	+	+	+/−	[21]
AH681	Chz	Avirulent	pig	Healthy	+	+	–	[18]
HN136	Chz	Avirulent	pig	Healthy	+	+	–	[18]
ST1	1	Avirulent	pig	Healthy	+	+	–	[27]
HA0609	2	Avirulent	pig	Healthy	+	+	–	[28]
NSUI002	2	Avirulent	pig	Healthy	+	+	–	[33]
05HAS68	2	Avirulent	pig	Healthy	+	+	–	[34]
NSUI060	2	Avirulent	pig	Healthy	+	+	–	[35]
YB51	3	Avirulent	pig	Healthy	+	+	–	[36]
6407	4	Avirulent	pig	Healthy	+	+	–	[37]
D9	7	Avirulent	pig	Healthy	+	+	–	[27]
D12	9	Avirulent	pig	Healthy	+	+	–	[38]
TL13	16	Avirulent	pig	Healthy	+	+	–	[39]
LS9N	–	Avirulent	+	Healthy	+	+	–	[40]
90–1330	–	Avirulent	+	Healthy	+	+	–	[41]
DN13	9	Avirulent	pig	Healthy	+	+	–	[42]

**Fig. 4:**
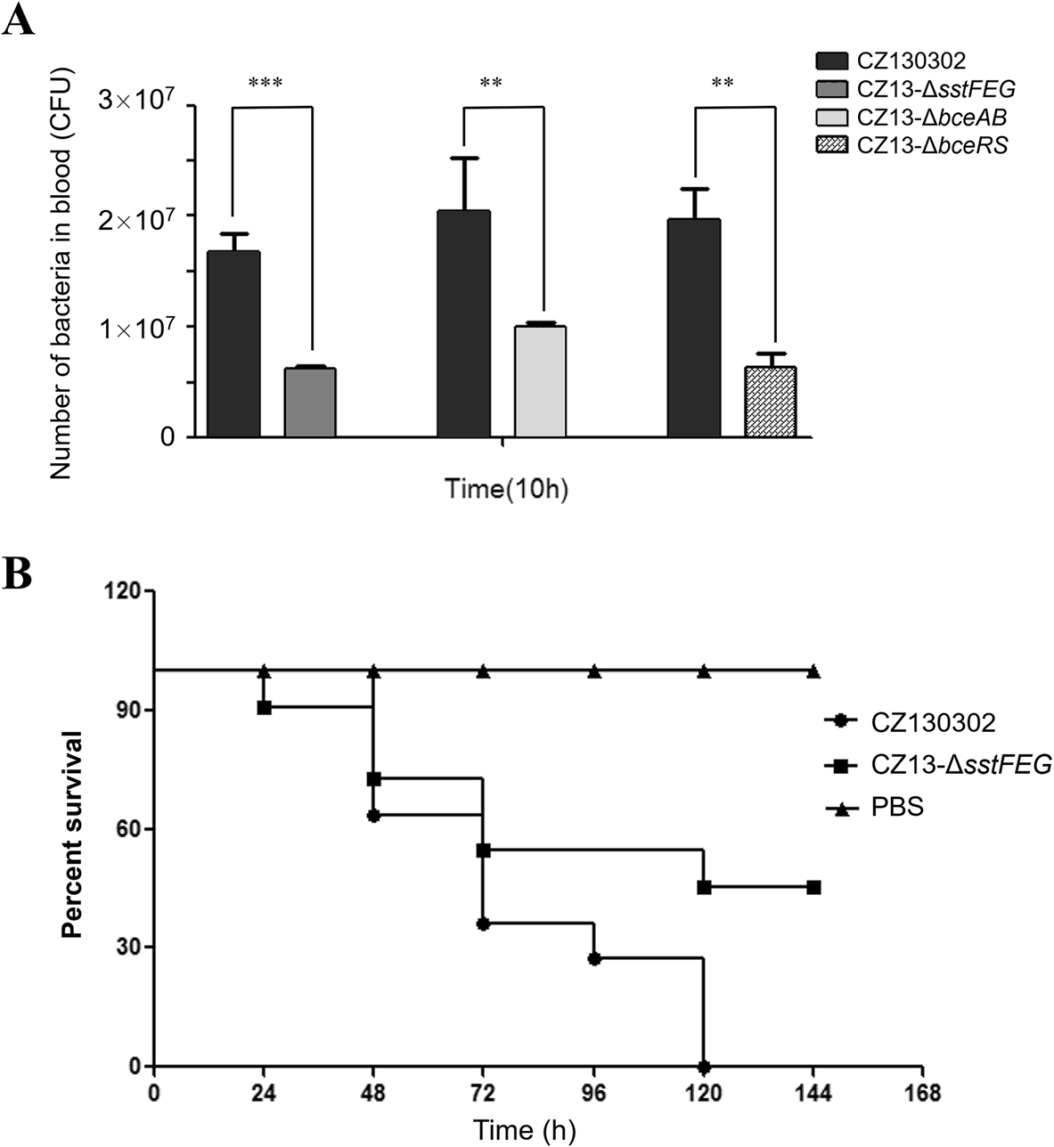
Bacterial competitive colonization and virulence analysis in vivo. **a** The competitive test was carried out to compare the colonization between wild-type and mutant strains in mouse infection model. The bacterial loads of mutant strains were decreased by approximately two- or three-fold in comparison with the wild-type strain in blood after intraperitoneal challenge for 10 h, ** *P* < 0.01 and *** *P* < 0.001. **b** Survival curves of BALB/c mice infected with wild-type or mutant strains. Six-week-old female BALB/c mice were segregated into three groups and inoculated i.p. with 2.6 × 10^7^ cells/mouse. Mice infected with the vehicle solution (PBS) were used as controls and survival was monitored over a 7-d period. Data are expressed as the mean percentage of live animals in each group (*n* = 11), * *P* < 0.05

The colonization of *S. suis* requires to antagonize mucosal microflora and host immune clearance. Many bacterial species from mucosal microflora can secrete bacitracin to achieve competitive advantage for optimal survival. Furthermore, bacitracin was previously used as a growth-promoting supplement in animal feed. Here, we managed to test whether SstFEG efflux pump is required for *S. suis* original colonization. Indeed, the inactivation of SstFEG resulted in a 2- to 3-fold reduction in the bacterial loads compared to the wild-type strain in blood after intraperitoneal injection for 10 h (Fig. 4a), indicating that SstFEG mediating bacitracin tolerance are essential for *S. suis* to rapidly infect the host. As shown in Fig. 4b, mice infected with the wild-type strain CZ130302 had severe clinical symptoms and displayed 100% mortality on the fifth day after challenge. In contrast, the group infected with the mutant CZ13-△*sstFEG* strain displayed a 50% greater survival rate (5/11).

## Discussion

Thus far, the underlying mechanism of bacitracin resistance has been reported in *B. subtilis*, *S. mutans,* and *S. pneumoniae,* but not in *S. suis*. The present study shows that the network of bacitracin resistance in *Streptococcus* partially comprises TCS and ABC transporters, as revealed from the KEGG Pathway Database (Additional file 3). BceSR (TCS) and BceAB (ABC transporter) are ubiquitous and significantly influence the bacitracin resistance of *Streptococcus* spp. In this study, we identified a novel ABC transport module (CVO91___06470, 06465, and 06460) mediating bacitracin resistance and virulence in *S. suis*. The CVO91___06470 gene encodes an ABC transporter ATP-binding protein that shares approximately 42% protein homology with the BcrA system, contributing to bacitracin resistance in *B. subtilis*.

**Fig. 5:**
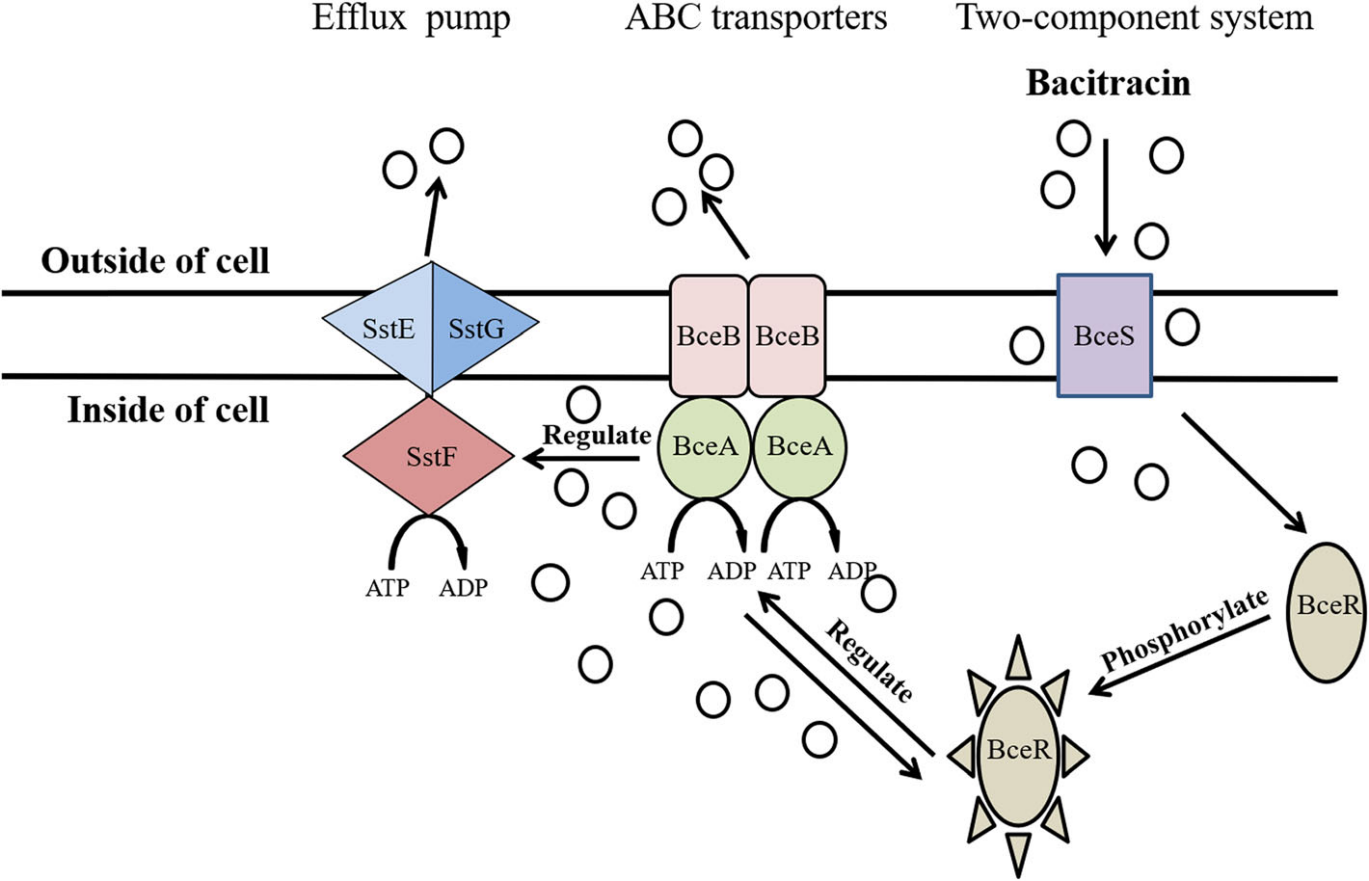
The underlying network regulating bacitracin sensing and resistance in *S. suis*. We supposed that when BceS senses extracellular bacitracin, it activates BceR via phosphorylation. Phosphorylated BceR can not only directly regulate BceAB to transport bacitracin, but also regulate SstFEG efflux pump to further export bacitracin

Interestingly, these three genes (designated as *sstFEG*) are coincidentally located upstream of the four genes encoding well-known BceAB and BceSR homologs (Fig. 1a). Bacitracin sensing depends on the BceRS signal transduction system and the BceAB transporter as a co-sensor. Jing Ouyang et al [12] reported that phosphorylated BceR upregulates the positively regulated expression of the *bceABRS* operon. In this study, we proposed that BceAB, BceRS, and SstFEG may constitute a seven-component system to regulate the bacitracin efflux optimally. Our results indicated that the bacitracin tolerance mediated by SstFEG is not only independent of the BceAB transporter, but also regulated by the TCS BceSR. Furthermore, *sstFEG* and *bceAB* are controlled by different operons, but the deletion of *bceAB* caused significant downregulation of *bceRS*, which decreased the bacitracin resistance mediated by SstFEG by inhibiting the transcriptional activation. In contrast, the deletion of *sstFEG* could not regulate the BceAB and BceSR systems for sensing bacitracin. A model for this regulatory pathway of bacitracin resistance in *S. suis* is proposed (Fig. 5).

Except for the bacitracin resistance, antibiotic susceptibility testing indicated that strain CZ130302 exhibits a more extensive drug resistance pattern than that of other *S. suis* strains. Comparative genomics studies have reported that the genomes of newly isolated CZ130302 harbor at least 45 ISs, including the novel 82-kb integrative conjugative element ICE*Ssu*CZ130302 [17], which may increase the potential horizontal gene transfer from different bacterial species or genera. Further studies need to explore how and why strain CZ130302 acquired numerous exogenous antibiotic resistance genes and integrated into a chromosome, especially for newly identified SstFEG efflux pump.

Given that the SstFEG never been reported in any other opportunistic or commensal bacteria species, we speculated that its encoding genes may be transferred from pathogenic bacterial strains. To further examine the correlation between the virulence of *S. suis* strains and SstFEG, genomic data of 34 *S. suis* strains from GenBank were investigated. Consequently, SstFEG are harbored by almost all virulent strains isolated from the patients and diseased pigs, but not in the avirulent strains isolated from healthy pigs. To further explore the potential roles of SstFEG in *S. suis* virulence, BALB/c mouse infection model was employed for challenge tests [44]. Indeed, the pathogenicity of the mutant strain deleted *sstFEG* was significantly decreased.

## Conclusions

This study reports a novel membrane transporter module SstFEG, which functions as not only an efflux pump for bacitracin resistance, but also a virulence-related protein involved in *S. suis* pathogenicity.

## Methods

### Bacterial strains, plasmids, and culture conditions

*S. suis* strain CZ130302 from the novel serotype Chz was isolated from a diseased piglet in Changzhou, China [18, 45]. Plasmid pSET-4S with Spc resistance gene was generously provided by Professor Daisuke Takamatsu. Plasmid pUC19 was maintained in the OIE Reference Laboratory for Swine Streptococcosis. *S. suis* were cultured in Todd Hewitt Broth (THB, BD) or agar comprising 6% (v/v) sheep blood at 37 °C and 5% CO_2_. *E. coli* strains were cultured in Luria-Bertani (LB, BD) medium at 37 °C supplemented with 100 μg/mL Spc (Sigma) per requirement for *S. suis* and 50 μg/mL Spc or 100 μg/mL ampicillin (Amp, Sigma) for *E. coli*. Different types of antibiotics, especially bacitracin (Bac, Sigma), were used to determine the minimum inhibitory concentration. In total, 100 μg/mL lysozyme (Lzm, Sigma) and 1 μg/mL vancomycin (Van, Sigma) were used for phagocytosis assays.

### Antimicrobial susceptibility assays

In accordance with the standardized methods per the Clinical and Laboratory Standards Institute (CLSI) guidelines (2015), the minimum inhibitory concentrations (MICs) of different types of antibiotics, including beta-lactams, aminoglycosides, tetracyclines, amide alcohols, macrolides, lincosamides, polypeptides, and fluoroquinolone against *S. suis* CZ130302 were determined. In brief, the strains were diluted 1000-fold into Cation Adjusted Muller Hinton Broth (CAMHB) with lysed horse serum (2.5% v/v) and cultured to an optical density at 600 nm (OD_600_) of approximately 0.5; thereafter, 180 μL of the culture was inoculated into the first vertical well, while 100 μL was inoculated in the other wells. Different initial antibiotic concentrations in 20 μL were placed in the first well and mixed, and 100 μL of the mixture from the first well was transferred to the subsequent well. This was repeated and as an analogy until the last well. Subsequently, cultures were incubated at 37 °C and 5% CO_2_ for 20 h. CZ130302 was tested through a serial dilution from 10^− 1^ CFU to 10^− 7^ CFU, and the THB plates with a 2-fold dilution of bacitracin, initiating at 64 μg/mL. All experiments were performed in triplicate. The *Streptococcus pneumoniae* strain ATCC 49619 was used as a control to ensure the reliability of tested data.

### Prediction and distribution of bacitracin-resistant genes in *Streptococcus suis*

The complete genome sequence of *S. suis* strain CZ130302 was obtained from NCBI (https://www.ncbi.nlm.nih.gov/, GenBank: CP024974.1). Antibiotic Resistance Genes Database (ARDB, http://ardb.cbcb.umd.edu/) was used to predict resistance genes in the genome of *S. suis*. KEGG PATHWAY Database (http://www.genome.jp/kegg/pathway.html) was used to extract previously reports pathways underlying bacterial bacitracin resistance and the distribution of resistance genes in *S. suis* exhibited in the NCBI database. In addition, Rockhopper software was used to predict the operon, transcription start site (TSS), and transcription termination sites (TTS) of CZ130302 in accordance with the distribution of reads in the genome.

### Construction of gene deletion mutants

To investigate the contribution of predictable genes, a series of deletion mutants was constructed via natural DNA transformation instead of the traditional method with pSET-4 s because this method was not suitable for *S. suis* CZ130302. Primers used to construct and confirm the mutants are enlisted in Additional file 4. The construction strategy for *sstFEG* deletion mutant strain is shown in Fig. 1. In brief, fragment AB (574 bp) and CD (867 bp) were amplified from *S. suis* CZ130302, using primers *sst-A* (BamHI site at its 5′ end) and *sst-B*, *sst-C*, and *sst-D* (Sal I site at its 3′ end) as upstream and downstream of *sstFEG*, respectively. The *spc* sequence (1133 bp) with the promoter was amplified from plasmid pSET-4 s by primers *Spc-F* and *Spc*-*R*. These three fragments were fused via PCR and ligated with pUC19 to form the recombinant plasmid pUC19-AB-Spc-CD. The CZ13-△*sstFEG* mutant was obtained via homologous recombination and resistance screening. Other mutant strains including CZ13-△*bceAB* and CZ13-△*bceRS* were constructed as previously described.

### Anti-pressure analysis of bacitracin

*sstFEG* mutant strains were cultured up to an optical density of approximately 0.7 at 600 nm (OD_600_). Thereafter, bacitracin was added at a high concentration. All bacteria were cultured for 8 h at 37 °C and 5% CO_2_ continuously, followed by plating of serial ten-fold dilutions on THB agar to enumerate bacteria at each hour. The trial was performed in triplicate, and the results were determined using the following formula: CFUs of viable bacteria at each hour − CFUs of original bacteria.

### qRT-PCR analysis

To analyze the expression levels of related genes and their interactions, total RNA was isolated from CZ130302 and mutant strains upon culturing to an optical density at 600 nm (OD_600_) of 0.6 in THB broth, with or without 2 μg/mL bacitracin, using RNAiso Plus (Takara, Japan) in accordance with the manufacturer’s instructions. When bacterial strains were cultured with bacitracin, the *Streptococcus pneumoniae* strain ATCC 49619 was used as control. The resulting RNA was then treated with gDNA Eraser to remove genomic DNA and further converted to cDNA via reverse transcription (RT), using PrimeScript™RT reagent Kit (Takara). cDNA samples were synthesized using RNA species harvested from three independent cultures of each strain. Real-time quantitative PCR assays were performed in triplicate with the method of SYBR Green detection. Primers for qRT-PCR analysis are enlisted in Additional file 4. The relative amount of target gene mRNA was normalized to the housekeeping gene *parC* [46]. The relative fold change was calculated by the threshold cycle (2^-△△^*C*_*T*_) method [47]. Each assay was performed in duplicate in three independent experiments.

### Animal experiments

A competitive test was performed to compare the colonization ability between wild-type and mutant strains in mice. Prior to this experiment, all mice were fed with bacitracin in water to activate bacterial genes. Eighteen female 6-week-old BALB/c mice were equally segregated into three groups. The wild-type and mutant strains were cultured until the OD_600_ was approximately 0.8, and the density of each strain was adjusted to 1.3 × 10^8^ CFU in PBS. Thereafter, the wild-type and different mutant strains were respectively mixed in a 1:1 ratio and challenged intraperitoneally with 200 μL/mouse. After 10 h, blood was sampled from each mouse, followed by plating serial five-fold dilutions on THB agar, containing 50 μg/mL kanamycin with or without 100 μg/mL Spc to distinguish and enumerate bacteria. In addition, the virulence of mutant CZ13-△Sst FEG strain was assessed in the BALB/c mouse model of infection. Thirty-three female 6-week-old BALB/c mice were equally segregated into three groups and challenged intraperitoneally with 200 μL/mouse at approximately 2.6 × 10^7^ CFU (10 × LD_50_) in PBS. Mice infected with the vehicle (PBS) were used as controls. Mortality was monitored every 8 h for 7 d. All experiments were performed in triplicate. All animals used in this study were humanely euthanized by carbon dioxide asphyxiation in an airtight box.

### Statistical analyses

GraphPad Prism version 5 is used to analyze and plot the data. Student’s *t*-test (unpaired) was performed to determine differences between the means of the two samples. Differences with a *P* value of < 0.05 were considered significant, and a *P*-value of < 0.01 was considered greatly significant.

### Ethics statement

Six-week-old female germfree BALB/c mice were purchased from the Comparative Medicine Center of Yangzhou University. Animal experiments were carried out in the Laboratory Animal Center of Nanjing Agricultural University and approved by Laboratory Animal Monitoring Committee of Jiangsu Province, China [Permit number: SYXK (SU) 2017–0007].

## Supplementary information

**Additional file 1.** Identification of mutant strains via PCR.

**Additional file 2. **Comparison of growth curves between wild-type CZ130302 and mutant strain CZ13-△*sstEFG*.

**Additional file 3.** Genes involved in bacitracin transport.

**Additional file 4.** Primers used in this study.

## Data Availability

All data generated or analysed during this study are included in this published article and its supplementary information files.

## Abbreviations

CLSI: Clinical and Laboratory Standards Institute
LB: Luria-Bertani
MICs: The minimum inhibitory concentrations
SS: *Streptococcus suis*
TCS: Two-component system
THB: Todd Hewitt Broth
TSS: Transcription start site
