# Supplementary material for: Bacitracin resistance and enhanced virulence of Streptococcus suis via a novel efflux pump
Source: BMC Vet Res. 2019 Oct 28;15:377. doi: 10.1186/s12917-019-2115-2 (PMC6819616; doi:10.1186/s12917-019-2115-2)
Supplement: Supplementary file 1 — Additional file 1. Identification of mutant strains via PCR. [file 12917_2019_2115_MOESM1_ESM.docx]

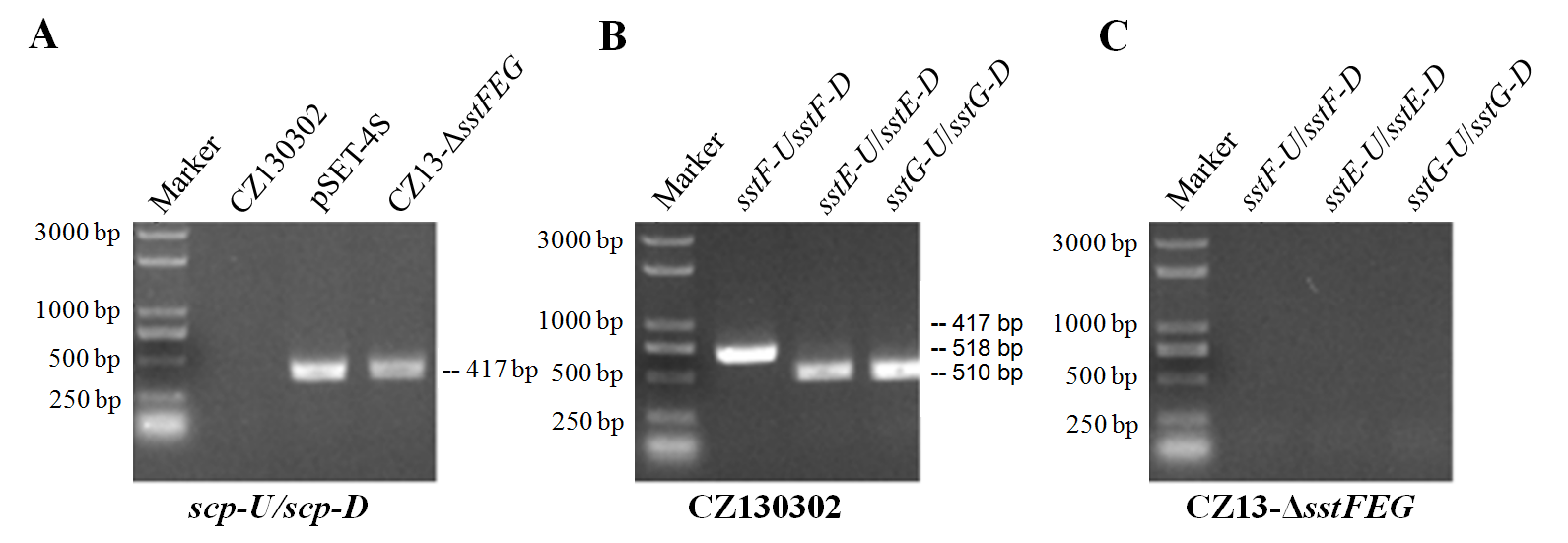


**Additional file 1:** **Identification of mutant strains via PCR.** Target genes were replaced by *Spc* gene by allelic exchange, with subsequently sub-cultured bacteria displaying stability. All PCR products showed the expected bands upon electrophoresis. A 417-bp fragment of the *Spc* gene was amplified using primers *Spc-U*/*Spc-D* in the mutant strain; however, no bands were detected in the parental strain (A). The specific DNA bands with the expected size could be detected by agarose gel electrophoresis. (B and C).
