# Supplementary material for: Bacitracin resistance and enhanced virulence of Streptococcus suis via a novel efflux pump
Source: BMC Vet Res. 2019 Oct 28;15:377. doi: 10.1186/s12917-019-2115-2 (PMC6819616; doi:10.1186/s12917-019-2115-2)
Supplement: Supplementary file 2 — Additional file 2. Comparison of growth curves between wild-type CZ130302 and mutant strain CZ13-△sstEFG. [file 12917_2019_2115_MOESM2_ESM.docx]

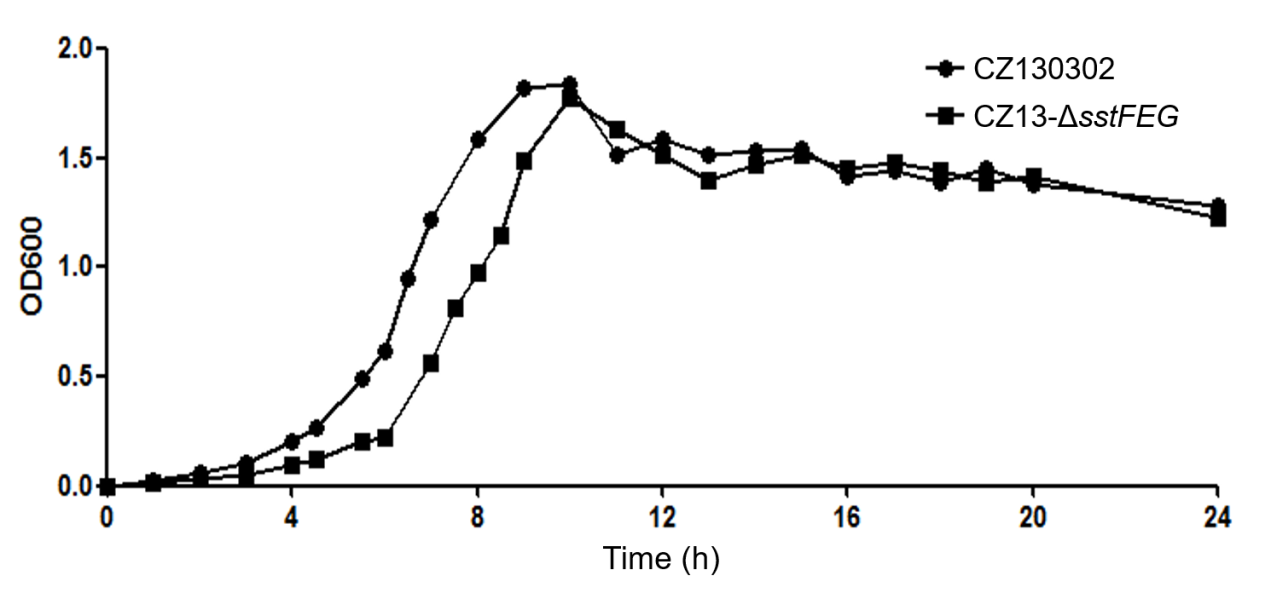


**Additional file 2:** **Comparison of growth curves between wild-type CZ130302 and mutant strain CZ13-△*sstEFG*.** Growth rates were monitored at each time point under the same conditions *in vitro*. Except for a slight difference during the log phase, the trend and the highest points of the two curves were essentially the same.
