# Supplementary material for: Bacitracin resistance and enhanced virulence of Streptococcus suis via a novel efflux pump
Source: BMC Vet Res. 2019 Oct 28;15:377. doi: 10.1186/s12917-019-2115-2 (PMC6819616; doi:10.1186/s12917-019-2115-2)
Supplement: Supplementary file 3 — Additional file 3. Genes involved in bacitracin transport. [file 12917_2019_2115_MOESM3_ESM.docx]

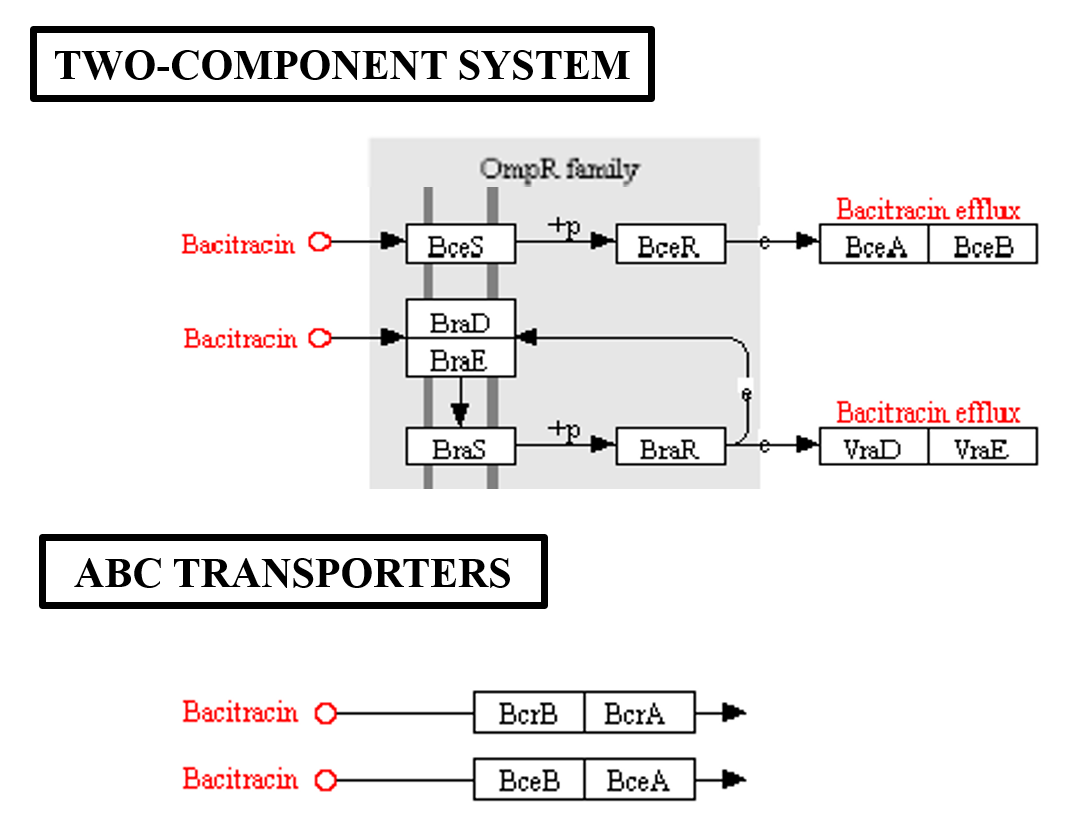


**Additional file 3: Genes involved in bacitracin transport.**

The major regulators of bacitracin transport are the two-component systems (BceSR and BraSR) and ABC transporters (BceAB and BcrAB) in accordance with the KEGG PATHWAY Database.
