# Supplementary material for: Bacitracin resistance and enhanced virulence of Streptococcus suis via a novel efflux pump
Source: BMC Vet Res. 2019 Oct 28;15:377. doi: 10.1186/s12917-019-2115-2 (PMC6819616; doi:10.1186/s12917-019-2115-2)
Supplement: Supplementary file 4 — Additional file 4. Primers used in this study. [file 12917_2019_2115_MOESM4_ESM.docx]

**Additional file 1: PCR** primers used in this study.

| Name | Primer Sequences（5’-3’）^a^ | Size(bp) |
| --- | --- | --- |
| *spc-F* | TAGTGTTCGTGAATACATGTT | 1133 |
| *spc-R* | TTTTCTAAAATCTGATTACCA |  |
| *sst-A* | CGCGGATCCCAGATAGCTCAAAAGGTCGTT | 574 |
| *sst-B* | TCACGAACACTAAAACCTTTTACAATTTCATGT |  |
| *sst-C* | GATTTTAGAAAATACTCCTAATTCCTTATTCAC | 867 |
| *sst-D* | GCGTCGACGATACGACTGACTATGTTCAC |  |
| *bceAB-A* | CGCGGATCCTATATATTTCTTTCCGATGCC | 558 |
| *bceAB-B* | TCACGAACACTAGCCTTGTGCGAGATTTCTTGC |  |
| *bceAB-C* | GATTTTAGAAAATTCTTTAGCAAAATTAACTTG | 602 |
| *bceAB-D* | GCGTCGACAACTCTTGACATGCTAAAGGC |  |
| *bceRS-A* | CGCGGATCCATAAGGCGTAATCAAATCTGC | 574 |
| *bce**RS-B* | TCACGAACACTATTTTTTCTTTTGAAAGCGTTT |  |
| *bceRS-C* | GATTTTAGAAAAATTCTGCAAAAGCCAGGACAC | 577 |
| *bceRS-D* | GCGTCGACACAATGGTAATACCAAAGGAA |  |
| *spc-U* | CGTCGTATCTGAACCATTGAC | 417 |
| *spc-D* | CGGCATAAAGTTAATATAGAG |  |
| *sstF-U* | AGTTACTTGACTTTCGGATGC | 681 |
| *sstF-D* | ATCTACGGCTTTCTTGGAC |  |
| *sstE-U* | ATCAAGCAGTAAATGGCATAA | 518 |
| *sstE-D* | TTCCCAATTGCAGTTTGTGTG |  |
| *sstG-U* | TCATATCTTGCAATGCCCTAC | 510 |
| *sstG-D* | TCGACGGAACTCAATCTTTGG |  |
| *bceA-U* | CTGCATTGGCCGAGTGAGTTA | 531 |
| *bceA-D* | AGGTAGAAGAGGGCGAGTTTA |  |
| *bceB-U* | ACAAAGTGTAGGGTCGCCATA | 1361 |
| *bceB-D* | GTCATGACCGCCTTTCTC |  |
| *bceR-U* | CTTGGTTTCCGTAGGTGAA | 374 |
| *bceR-D* | AGAATTTCCGAGCTGTCAGTC |  |
| *bceS-U* | TAATCTGTGATAGGGCAAAGG | 400 |
| *bceS-D* | TTGATTGAAGCGGAGAAGG |  |

^a^ The underlined sequences are restriction enzyme sites
